# Flux estimation analysis systematically characterizes the metabolic shifts of the central metabolism pathway in human cancer

**DOI:** 10.3389/fonc.2023.1117810

**Published:** 2023-06-12

**Authors:** Grace Yang, Shaoyang Huang, Kevin Hu, Alex Lu, Jonathan Yang, Noah Meroueh, Pengtao Dang, Yijie Wang, Haiqi Zhu, Sha Cao, Chi Zhang

**Affiliations:** ^1^ Center for Computational Biology and Bioinformatics, Indiana University School of Medicine, Indianapolis, IN, United States; ^2^ Carmel High School, Carmel, IN, United States; ^3^ Park Tudor School, Indianapolis, IN, United States; ^4^ Department of Electrical and Computer Engineering, Purdue University, Indianapolis, IN, United States; ^5^ Department of Computer Science, Indiana University, Bloomington, IN, United States; ^6^ Department of Biostatistics, Indiana University School of Medicine, Indianapolis, IN, United States; ^7^ Department of Medical and Molecular Genetics, Indiana University School of Medicine, Indianapolis, IN, United States

**Keywords:** cancer metabolism, flux estimation, glutaminolysis, TCA cycle, systems biology

## Abstract

**Introduction:**

Glucose and glutamine are major carbon and energy sources that promote the rapid proliferation of cancer cells. Metabolic shifts observed on cell lines or mouse models may not reflect the general metabolic shifts in real human cancer tissue.

**Method:**

In this study, we conducted a computational characterization of the flux distribution and variations of the central energy metabolism and key branches in a pan-cancer analysis, including the glycolytic pathway, production of lactate, tricarboxylic acid (TCA) cycle, nucleic acid synthesis, glutaminolysis, glutamate, glutamine, and glutathione metabolism, and amino acid synthesis, in 11 cancer subtypes and nine matched adjacent normal tissue types using TCGA transcriptomics data.

**Result:**

Our analysis confirms the increased influx in glucose uptake and glycolysis and decreased upper part of the TCA cycle, i.e., the Warburg effect, in almost all the analyzed cancer. However, increased lactate production and the second half of the TCA cycle were only seen in certain cancer types. More interestingly, we failed to detect significantly altered glutaminolysis in cancer tissues compared to their adjacent normal tissues. A systems biology model of metabolic shifts through cancer and tissue types is further developed and analyzed. We observed that (1) normal tissues have distinct metabolic phenotypes; (2) cancer types have drastically different metabolic shifts compared to their adjacent normal controls; and (3) the different shifts in tissue-specific metabolic phenotypes result in a converged metabolic phenotype through cancer types and cancer progression.

**Discussion:**

This study strongly suggests the possibility of having a unified framework for studies of cancer-inducing stressors, adaptive metabolic reprogramming, and cancerous behaviors.

## Introduction

Dysregulation of metabolic pathways is a hallmark of cancer (1, 2). In the past decades, new biotechnologies and experimental systems have advanced substantial knowledge of metabolic shifts and their functional roles in the oncogenesis process and the progression of cancer (3). Metabolic phenotypes of cancer and stromal cells and mechanistic insights into how the metabolic system is shifted along the coevolution of cancer and the tumor microenvironment (TME) have been discovered on different experimental systems, such as cancer cell lines, mouse models, patient-derived xenografts, or organoid models (3, 4). Despite a plethora of knowledge gained on the core components of metabolic pathways, there are still major gaps in our understanding of the integrated behavior and metabolic heterogeneity of cells in TME. Essentially, the metabolic behavior can be determined by different factors and vary dramatically from cell to cell and tissue to tissue due to their high plasticity, driven by the need to cope with various dynamic metabolic requirements and biochemical conditions (3).

The Warburg effect, characterized by a shifted flux ratio between aerobic and anaerobic respirations, is considered a common metabolic reprogramming mechanism in human solid cancer (5, 6). The further discovery of the glutaminolysis pathway elucidates the role of “fueling” for energy production and the biosynthesis of other metabolites such as amino acids, which also expanded the definition of central metabolism in cancer metabolism (7). In addition to these “common” metabolic shifts, variations in branches of the central metabolic pathway have been observed in different cancer types, including biosynthesis of nucleotide, biosynthesis of serine and glycine, Cori cycle and gluconeogenesis, malate shuttle and aspartate metabolism, redox balance and glutathione metabolism, biosynthesis of fatty acids, synthesis of immune-metabolite 2-hydroxyglutarate from a-ketoglutarate, and cytosolic metabolism of glutamine and glutamate (8–11). Notably, the majority of these analyses are made on cell lines or mouse systems, which cannot mimic the dysbalanced redox, pH, and oxygen levels. In addition, the nutrient supplies of the experimental system also differ from those of real cancer tissue, as both glucose and glutamine are always sufficiently provided under experimental conditions, while their availability levels and ratios heavily shift through cancer and determine metabolic phenotypes. In addition, recent spatial techniques suggest the heterogenous distribution of metabolic stresses in real cancer tissues, which promotes metabolic competition and coadaptation between cancer and stromal cells (12). All the evidence suggests that the experimental systems under normal physiological conditions have drastically different biochemical characteristics compared to the TME of human cancer (13).

We would like to point out that the observations made in experimental systems may unnecessarily reflect the metabolic and nutrient-partition activities in human cancer tissues. For example, a recent study presented that myeloid cells consume the highest amount of glucose per cell in mouse tumor tissues, followed by T cells and tumor cells (14). To the best of our knowledge, numerous analyses of metabolic variations have been conducted on omics data collected from cancer tissue samples, but explicit analysis that mechanically estimates and quantifies metabolic shifts are lacking. We have recently developed a new graph neural network-based method, namely single-cell flux estimation analysis (scFEA), to predict metabolic flux by using single-cell transcriptomics data (14).

To provide an unbiased and comprehensive characterization of the landscape of metabolic changes in human solid cancer, we conducted a systematic evaluation of metabolic reprogramming and characteristics *via* computational analysis of pan-cancer transcriptomics data. We first reconstructed the central metabolism pathway by including glucose, glutamine, glutamate, and glutathione metabolism and six branches of the central metabolism network at subcellular resolution. We modified the scFEA method to fit the analysis of The Cancer Genome Atlas (TCGA) pan-cancer tissue transcriptomics data. Our analysis revealed distinct metabolic variations and shifts through different cancer types. As the Warburg effect has been identified in almost all analyzed cancer tissues, we did not see a significant contribution of glutaminolysis in fueling the TCA cycle. We identified that (1) normal tissues have distinct metabolic phenotypes, (2) cancer types have drastically different metabolic shifts, and (3) the different shifts that happened to tissue-specific metabolic phenotypes result in a converged metabolic phenotype through cancer types and cancer progression. Our analysis brought novel insights into the understanding of metabolic shifts in human cancer. Cancer and tissue type-specific metabolic shifts and the resulting convergent metabolic phenotype suggested the necessity of a personalized therapeutic strategy or nutrient and diet design for targeting metabolism in cancer treatment.

## Results

### Reconstruction of central metabolism pathway in the subcellular resolution

**Figure 1:**
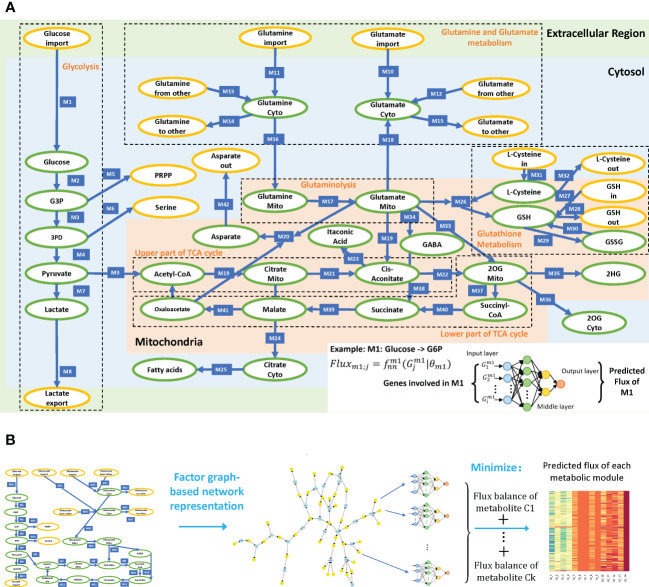
**(A)** Reconstructed central metabolism pathway. Intermediate and end metabolites are green and yellow labeled, respectively. The example on the bottom right showcases the neural network-based flux predictive model, which takes genes involved in module 1 (M1) and outputs predicted flux. **(B)** Analysis framework of scFEA.

To comprehensively evaluate the variations of energy metabolism in cancer, we collect the central metabolism network and its branches from the KEGG database and manually curate the reaction information from literature data based on our previously curated metabolic network (14). As subcellular compartments have different levels of enzymes, substrates, biochemical characteristics, and kinetic parameters, subcellular localization information of reactions is needed to accurately assess their stoichiometric relations. **Figure 1B** illustrates the reconstructed central metabolism network, including 42 reaction modules, 27 intermediate metabolites, 15 end metabolites, and 320 genes in the cytosol, mitochondria, and extracellular regions. The reconstructed central metabolism network includes six major pathways, namely glycolysis, upper and lower parts of tricarboxylic acid (TCA) cycle, glutaminolysis, glutamine and glutamate metabolism, and glutathione metabolism (15), and six minor branches, namely glyceraldehyde 3-phosphate (G3P) to nucleotide synthesis, 3-phospho-d-glycerate (3PD) to serine synthesis, aspartate–malate shuttle, mitochondrial citrate fueling of fatty acid synthesis, transport of 2-oxoglutarate (2OG) to cytosol, and transformation of 2OG to 2-hydroxyglutarate (2HG) (9).

### Estimation of sample-wise metabolic flux and metabolite abundance of central metabolism

We modified our recently developed method, scFEA, to enable the application of tissue transcriptomics data (14) (see details in **Methods**). scFEA models metabolic fluxes in a tissue based on gene expression data from a large number of samples by assuming (1) the total influx of each metabolite is approximately the same as its total outflux, which is constrained by a quadratic loss over the metabolic network; and (2) changes in the rate of each reaction can be modeled as a (nonlinear) function of changes in the expression levels of genes involved in the reaction and its neighbors, which could be modeled by a neural network. Note that assumption (1) is generally true unless some major in-/outflux for a metabolite is not considered. Assumption (2) is a combination of three simpler assumptions: (1) the concentration of an enzyme is a function of reaction rate that could be well supported by Michaelis–Menten equation; (2) the concentration of an enzyme is also a (nonlinear) function of the expression level of its encoding genes, with both functions being invariant across different samples of different cancer types; and (3) there exists a latent nonlinear relation between the concentration of metabolites and the genes involved in its transport and relevant reactions. Both assumptions (1) and (2) are supported by published studies (16–18). Intuitively, one can think of this model (for each reaction) as an integrated Michaelis–Menten model, whose parameters and nonlinear form are implicitly estimated by a neural network using a large number of available gene expression data (**Figure 1A**). The detailed formulation and parameters of the scFEA method utilized in this study are available in Methods.

**Figure 1B** outlines the flowchart of scFEA, with further details given in Methods. scFEA models the metabolic flux of each module using a three-layer, fully connected neural network of genes involved in the module, which minimizes the total imbalance of the intermediate substrates across all tissue samples. For the central metabolism network with K=42 modules and # genes as the average number of genes encoded in each reaction module, there are 12×K×(# genes) unknowns to be estimated, where 12 is determined by the neural network architecture. On the other side, there are K×N constraints, where K=27 and N=5,253 are the numbers of intermediate substrates and samples, respectively. As K×N≫12×K×(# genes) in  this study, the large number of samples in the utilized TCGA pan-cancer data enables sufficient statistical power to reliably estimation of the unknowns.

### Validation and application of scFEA in predicting the flux of the reconstructed central metabolism network

We have previously validated scFEA on glycolysis and TCA cycle pathways by applying the method on our in-house-generated scRNA-seq data of 88 patient-derived pancreatic cancer cell lines, Pa03c, and matched metabolomic profiling under two conditions: knockdown of APEX1 (APEX1-si) and scrambled control (sc) under hypoxia (14). In this study, we first applied scFEA to this scRNA-seq data and metabolomic profile to validate scFEA on the reconstructed central metabolism pathway. As scFEA has been previously validated over simplified central metabolism data, the goal of this validation is to confirm that scFEA can capture the metabolic changes on the newly reconstructed central metabolism pathway. We first examined the predicted metabolomic variation trend of the intermediate metabolites in Pa03c cells and compared the results with metabolomic profiling. Four metabolites of the glutaminolysis pathway, namely glutamine, glutamate, malate, and fumarate, that were not covered by our past analysis were examined here. We have seen that the scFEA-predicted abundance change of these four metabolites, in both cytosol and mitochondria, is consistent with the experimentally measured metabolomic changes (**Supplementary Figure S1B**). Specifically, glutamine and glutamate show a slight but insignificant increase in the APEX1-si condition compared to controls, while malate and fumarate show significantly decreased abundances.

APEX1 plays a central role in the cellular response to oxidative stress (19). scFEA predicts increased flux of the TCA cycle, decreased glycolysis in normoxic cells than hypoxic cells, and increased glutathione (GSH) to glutathione disulfide (GSSG) in APEX knockout cells. These observations match the experimentally observed metabolic changes in the Pa03c cells in our recent studies, including (1) the knockdown of APEX1 results in increased oxidative stress and cell death; (2) hypoxia triggers increased glycolytic activity and lactate production, (3) decreased TCA cycle; and (4) insignificantly changed glutamine metabolism in Pa03c cells (20). These observations demonstrated that the scFEA prediction can capture the major variations in the reconstructed central metabolism network under different biochemical conditions.

We further applied the modified scFEA on TCGA pan-cancer transcriptomics data of 11 cancer and subcancer types having matched adjacent normal controls, namely breast cancer (BRCA) luminal, her2-, and triple-negative (TNBC) subtypes, colon adenocarcinoma (COAD), head and neck cancer (HNSC), kidney renal clear cell carcinoma (KIRC), kidney renal papillary cell carcinoma (KIRP), lung adenocarcinoma (LUAD), prostate adenocarcinoma (PRAD), stomach adenocarcinoma (STAD), and thyroid carcinoma (THCA), totaling 5,253 samples (see details in Methods). Notably, scFEA predicts sample-wise in-/outflux for each intermediate metabolite. Hence, the relative abundance change of the intermediate metabolites can be estimated by the difference between their in- and outflux in each sample.

### A pan-cancer-level evaluation of metabolic variations of the central metabolic pathway and its branches

**Figure 2:**
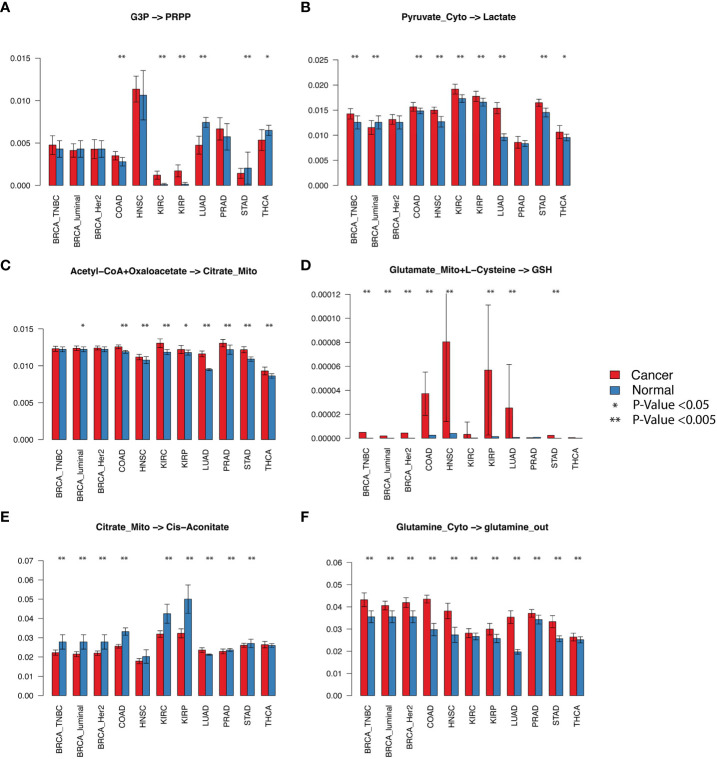
Selected fluxomic changes in cancer vs normal. The six panels listed the predicted metabolic flux of **(A)** the pentose phosphate module, **(B)** lactate production, **(C)** acetyl-CoA to citrate in the TCA cycle, **(D)** glutathione biosynthesis, **(E)** citrate to cis-aconitate, **(F)** glutamine metabolism in the analyzed cancer types. The x-axis and y-axis represent cancer types and predicted flux, respectively.

We first evaluated the flux changes between cancer and adjacent normal tissues. Our analysis suggested that the flux of glycolytic pathway (*p*< 0.001 in 10 out of 11 analyzed cancer types. Here, the same representation of the number of significantly increased or decreased pathways is used for other reaction modules), lactate production (seven of 11), lactate export (nine of 11), biosynthesis of glutathione (eight of 11), glutamate (seven of 11) and glutamine import (seven of 11), and glutamine (10 of 11), glutamate (eight of 11), and aspartate (six of 11) metabolism to other amino acids in the cytosol are consistently increased in cancer *vs*. normal tissues in almost all examined cancer types (**Figures 2A–D**; **Supplementary Figure S1A**; detailed *p*-values are given in **Supplementary Table S1**). We also observed a consistent decrease in the flux of fatty acid biosynthesis (five of 11), glutathione (six of 11) to other amino acids, enzyme-catalyzed glutathione to GSSG (six of 11), and cysteine metabolism (five of 11) (**Figures 2E, F** **;** **Supplementary Figure S1A**). The module from citrate to *cis*-aconitate (seven of 11) is consistently decreased, while the module from oxaloacetate to citrate (seven of 11) is increased in the upper part of the TCA cycle. The variations of the lower part of the TCA cycle differ through cancers. Although the glutamine and glutamate metabolism show distinct variations in cancer *vs*. normal tissues, we did not see a significant difference in glutaminolysis, i.e., glutamate to 2OG, gamma-aminobutyric acid (GABA), and succinate in mitochondria. We also did not observe a significant increase in the lower part of the TCA cycle. In addition, multiple cancer types tend to have decreased succinate to malate (three of 11) and malate to oxaloacetate (four of 11). **Supplementary Figure S2** illustrates the detailed flux changes of metabolic modules in the reconstructed central metabolism network.

**Figure 3:**
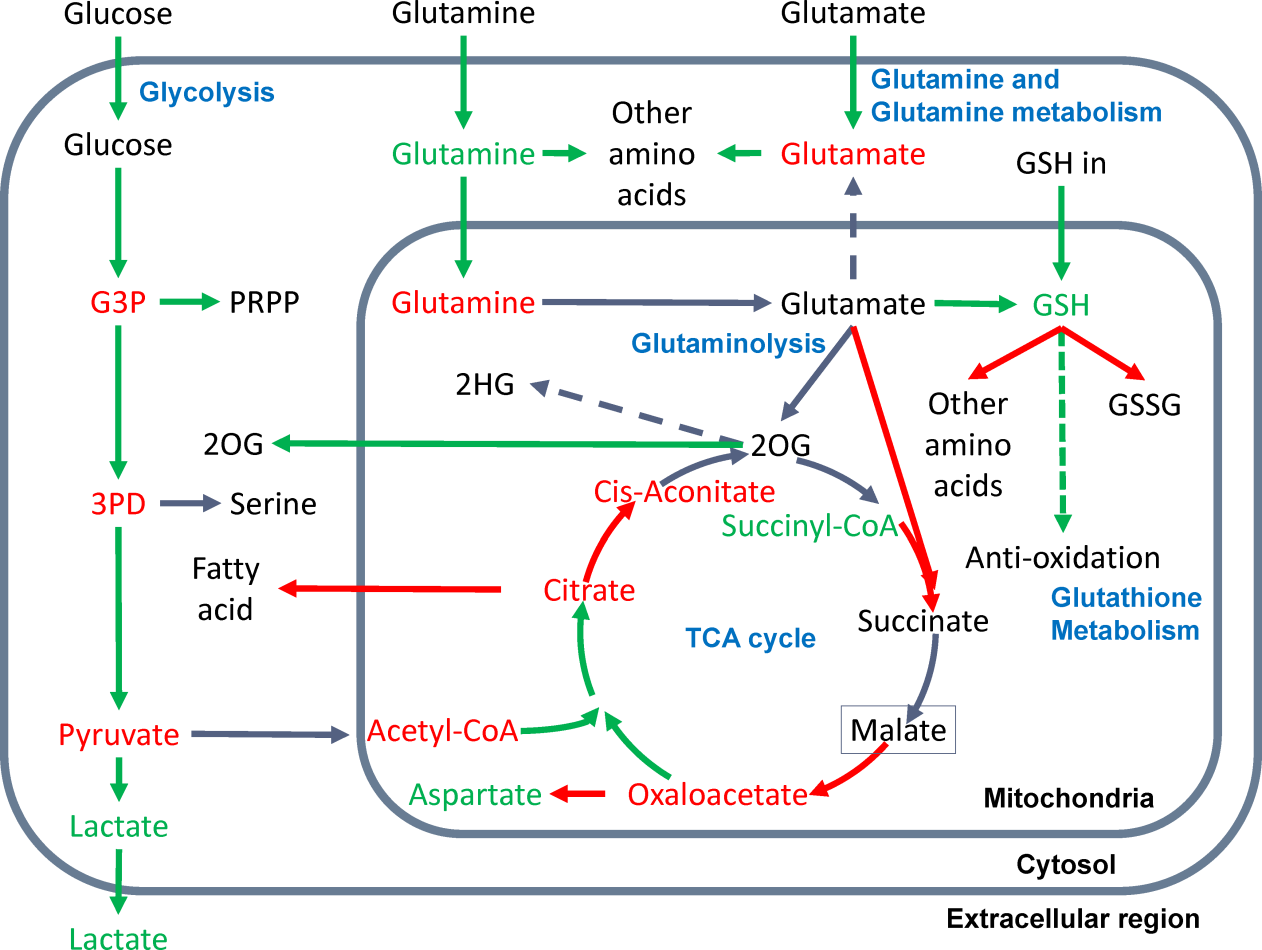
Metabolic variations in the central metabolism network were observed in this study. Increased or decreased flux of a reaction module is represented by a green or red arrow; accumulation or depletion of metabolites are green and red colored; the reactions and metabolites of inconsistent changes are grey and black colored, respectively. The dashed arrow suggests the predicted flux of the reaction is consistently zero or substantially low. The green dashed arrow of GSH to antioxidation suggests our inferred increased flux of GSH in antioxidation in cancer.

We also examined the predicted metabolomic changes in the central metabolism network. Notably, the flux balance assumption was considered in scFEA by a quadratic loss. The metabolomic change derived from predicted flux only reflects a trend of the variation of intermetabolites rather than their exact concentration change. We predicted consistently depleted glycolytic and TCA cycle intermediates, cytosolic glutamine (eight of 11), succinyl-CoA (five of 11), and cytosolic glutamate (10 of 11) in cancer *vs*. normal tissues, while the abundance of lactate (three of 11) and mitochondrial glutamine (five of 11) tends to be increased. **Figure 3** shows the detailed metabolic variations identified in this study. Below, we provide a few mechanistic interpretations of the observed metabolic variations.

#### Glycolysis

We observed increased glycolytic flux, depleted intermediates, and increased lactate production in almost all examined cancer types. These observations are consistent with the well-discussed Warburg effect. The only contradiction to experimental observations is that we did not see a significant increase in glucose uptake. As noted, the flux prediction is based on both gene expression and neighboring fluxes. Our previous studies confirmed the consistent upregulation of glucose transporters (21). The scFEA computes that the real glucose uptake is not increased, probably because the availability of glucose in TME is limited (12). We also identified that kidney cancer (KIRC) has the largest relative increase of glycolytic flux, while the TCA cycle is largely suppressed in KIRC (**Figure 2E**) but increases in lung cancer (LUAD). Our observation is consistent with a recent fluxomic experiment conducted on multiple patients’ cancer samples, which also observed a significant decrease in glucose oxidation in KIRC and activated glucose oxidation in LUAD (22).

#### TCA cycle

We observed that the majority of the TCA cycle reactions have a decreased flux in cancer *vs*. normal tissues, except for the first step of acetyl-CoA to citrate. The rate-limiting step from citrate to *cis*-aconitate is consistently decreasing. Most cancer types have decreased succinate to malate and malate to oxaloacetate. We also observed a consistent decrease of TCA cycle intermediates, except for the increased succinyl-CoA and varied changes of 2OG, succinate, and malate through cancer types. Our explanation is that the overall TCA cycle is suppressed, but the fueling from glutaminolysis relieves the depletion of TCA cycle intermediates at a certain level. However, in the TME of human cancer, the flux of carbon sources from glutaminolysis is not enough to fully refuel the decreased flux in the lower part of the TCA cycle.

#### Glutamate and glutamine metabolism

Cancer cells’ uptake of glutamate and glutamine is consistently increased in cancer *vs*. normal tissues. However, our flux analysis suggested that the majority of the glutamate and glutamine are utilized by the biosynthesis of other amino acids in cytosol rather than being transported into mitochondria to fuel the TCA cycle. We saw that the transport of glutamate from mitochondria to the cytosol is almost zero in all cancer types. The majority of the mitochondrial glutamate is utilized for glutathione biosynthesis than glutaminolysis.

#### Glutaminolysis

We did not observe a significant increase of glutaminolysis in cancer *vs*. normal tissues. However, the metabolomic change of intermediate substrates suggests that the fueling role of glutaminolysis truly relieves the largely depleted carbon source in the TCA cycle.

#### Glutathione metabolism

Our analysis included three input sources of GSH: biosynthesis from glutamate in mitochondria, biosynthesis in cytosol and transport into mitochondria, and reduction from GSSG. We also considered two outfluxes of GSH, namely biosynthesis of other amino acids and enzyme-catalyzed oxidation of GSH to GSSG. We observed an increased influx of GSH from glutamate and cytosolic biosynthesis, decreased outfluxes of GSH, and accumulated GSH abundance in cancer *vs*. normal tissues. As noted, our flux estimation only covers the enzyme-catalyzed reaction of GSH to GSSG, which does not include the reduction of reactive oxygen species generated under dysregulated metabolism. Based on the observations, we speculate that the majority of “accumulated” GSH predicted by scFEA is utilized for antioxidation, the flux of which was not included in the analysis.

#### Other branches

We also examined six branches of the central metabolic network. We observed an increase in nucleotide biosynthesis and transport of mitochondrial 2OG to cytosolic 2OG, decreased serine biosynthesis (except for KIRC and KIRP), fatty acid biosynthesis, and aspartate biosynthesis. We did not observe a significant flux from 2OG to immunosuppressive metabolite 2HG. A possible reason is that the reaction from 2OG to 2HG is catalyzed by mutated IDH1 or IDH2 enzymes, which are not covered by the current model (9).

The predicted fluxome and metabolomic changes are provided in **Supplementary Table S2**.

### Pan-cancer analysis suggests a convergent metabolic phenotype of human cancer

**Figure 4:**
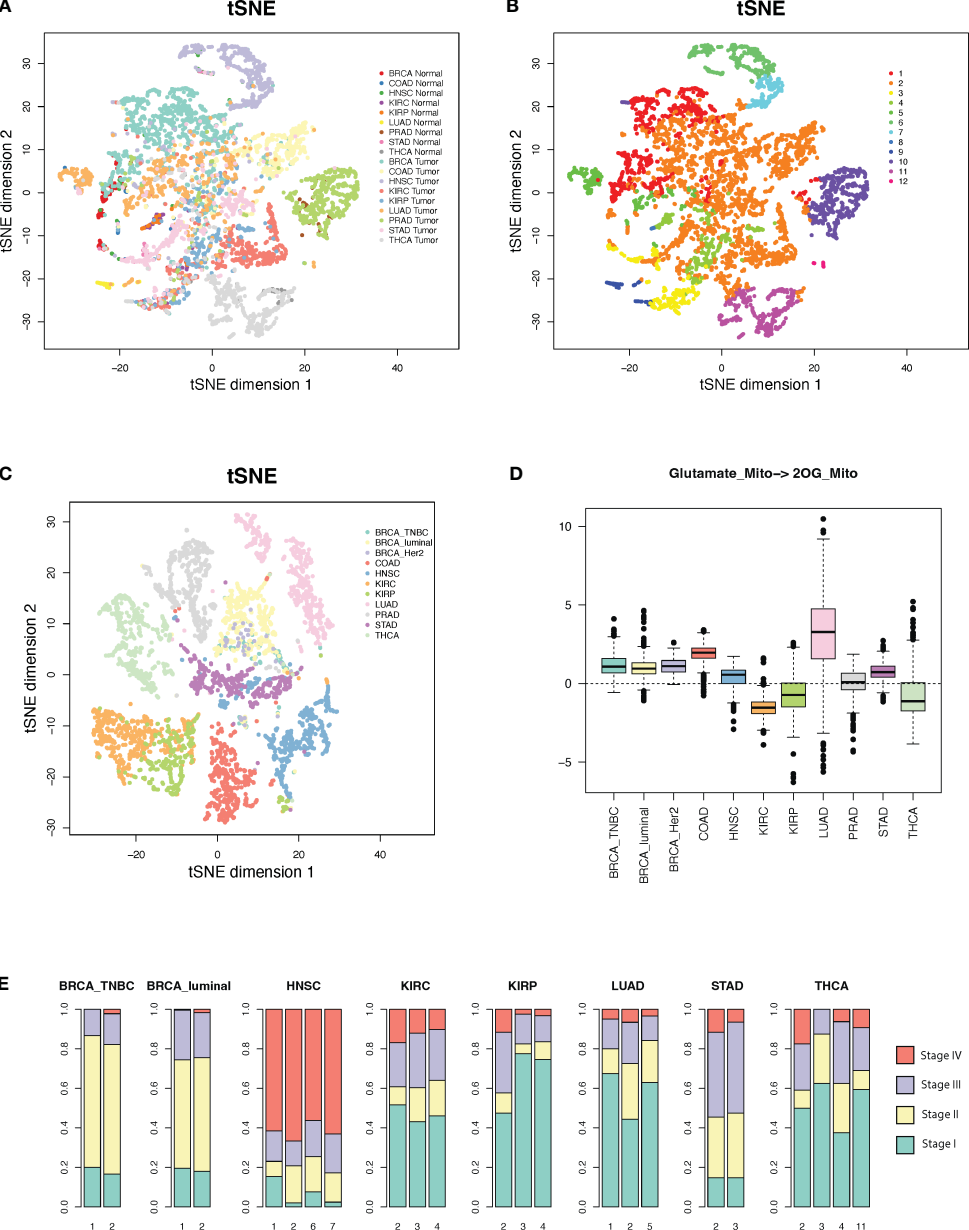
Metabolic phenotypes identified from pan-cancer analysis. **(A, B)** tSNE plots of cancer and normal tissues derived by using predicted flux. **(C)** tSNE plot of the cancer tissues derived by using flux change between cancer *vs*. adjacent normal controls. **(D)** Flux change between cancer *vs*. adjacent normal controls of the reaction of mitochondrial glutamate to 2OG. **(E)** Distribution of cancer stages in each cluster with respect to cancer types.

We conducted a pan-cancer comparative analysis of the predicted fluxome by using tSNE-based dimensional reduction. **Figure 4A** shows the 2D-tSNE plots of the analyzed samples from different cancer types derived by using the predicted flux distribution of the central metabolism network. **Supplementary Figure S3** lists the detailed distribution of each cancer and normal tissue type over the tSNE plot. We further conducted a K-nearest-neighbor-based clustering of the predicted flux based on their Euclidean distance and identified 12 clusters (**Figure 4B**). We have observed that (1) normal tissue types have distinctly varied metabolic phenotypes; (2) although some cancer types have different metabolic phenotypes compared to matched normal tissues, the fluxome of central metabolism in some cancer tissues, such as breast and colon cancer, is more similar to their matched normal tissues; (3) the fluxome of some cancer types are very similar, such as breast, colon, kidney and lung cancer; and (4) head and neck cancer, kidney renal papillary cell carcinoma, prostate cancer, and thyroid cancer have more distinct metabolic phenotypes.

Interestingly, we note a cluster (cluster 2 in **Figure 4B**) that consists of large sets of breast, colon, kidney, lung, stomach, normal breast, and colon cancer samples and small sets of head and neck, prostate, and thyroid cancer samples, while the other clusters are either tissue or cancer type specific. **Supplementary Table S3** lists the distribution of cancer and normal tissue types in each cluster. We further conducted a second tSNE analysis of the fluxome in cancer normalized by their adjacent normal controls. Specifically, we computed the Z-score of the flux of each module in a cancer sample against the flux of all normal controls of this cancer type and utilized the Z-score profile for tSNE analysis (**Figure 4C**). Our results suggest that (1) normal tissues have distinctly varied metabolic phenotypes, (2) cancer types have drastically different metabolic shifts (**Figure 4C**), and (3) the different shifts that happened to tissue-specific metabolic phenotypes result in a converged metabolic phenotype through cancer types (**Figure 4B**). **Figure 4D** illustrates the shifts in the flux of mitochondrial glutamate to 2OG, a key step in glutaminolysis, which shows a significant cancer-type specificity. Similarly, the majority of the reaction modules in the central metabolic network show different levels of shifts through cancer types.

We further checked the distribution of the cancer stage *vs*. the identified clusters. Cluster 2, which is the converged metabolic phenotype through multiple cancer types, is consistently enriched by cancers of more advanced stages for all the cancer types enriching this cluster (**Figure 4E**). Hence, with the progression of cancer, metabolic shifts tend to converge in this cluster. Further analysis suggests that this cluster has increased glycolytic activity, lactate production, decreased TCA cycle, slightly increased glutaminolysis, and more saved glutathione for potential antioxidation. However, this cluster does not have the highest change in such metabolic shifts compared to other clusters. Hence, we speculate that the central metabolic system, biochemical condition, redox balance, and demand of energy and substrates in the cancer tissues in this cluster are more tuned.

**Figure 5:**
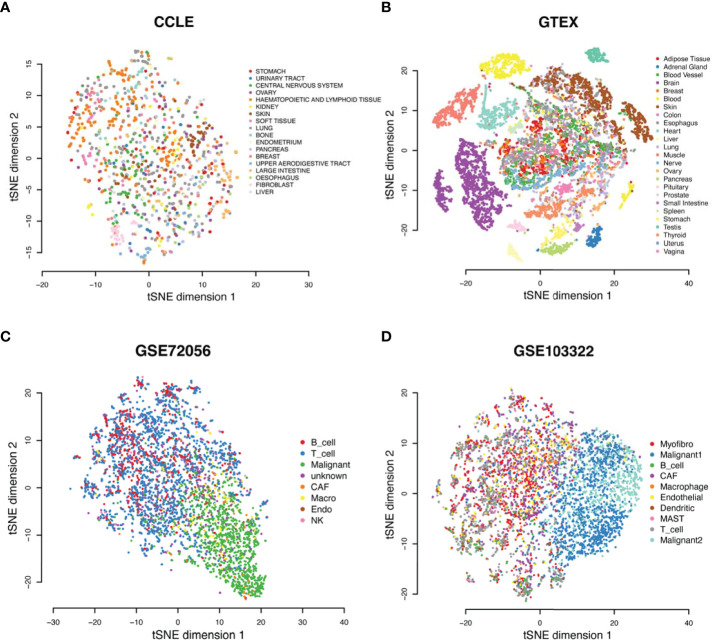
Metabolic phenotypes were identified from four independent datasets. **(A–D)** tSNE plots of **(A)** the CCLE cancer cell line, **(B)** GTEx normal tissue, **(C)** GSE72056 single cells from TME of melanoma, and **(D)** GSE103322 single cells from TME of head and neck cancer, derived by using the predicted fluxome of the central metabolism network.

To validate the observed converging trend of metabolic phenotypes through cancer types, we conducted the flux estimation analysis on Cancer Cell Line Encyclopedia (CCLE) data of 1,015 cell lines of 18 tissue types, GTEx data of 8,432 samples of 25 tissue types, scRNA-seq data of 4,486 single cells from eight cell types collected from the melanoma microenvironment (GSE72056), and scRNA-seq data of 5,902 single cells of 10 cell types collected from the head and neck cancer microenvironment (GSE103322). We conducted the same tSNE-based dimensional reduction and visualization by using the predicted fluxome of the four datasets. We observed that the metabolic phenotype of CCLE cancer cell lines is quite randomly distributed with respect to the tissue origins (**Figure 5A**), while the normal human tissues show distinct tissue type-dependent metabolic phenotypes (**Figure 5B**). Both scRNA-seq data suggest that the diversity of metabolic phenotypes of immune and stromal cells is higher than that of cancer cells (**Figures 5C, D** **)**. Specifically, cancer cells collected from different patient samples show lower divergence of the fluxome of central metabolism pathways compared to immune and stromal cells. Our analysis of these four independent datasets partially validated the converged trend of metabolic phenotypes of different cancer types observed on TCGA data.

**Figure 6:**
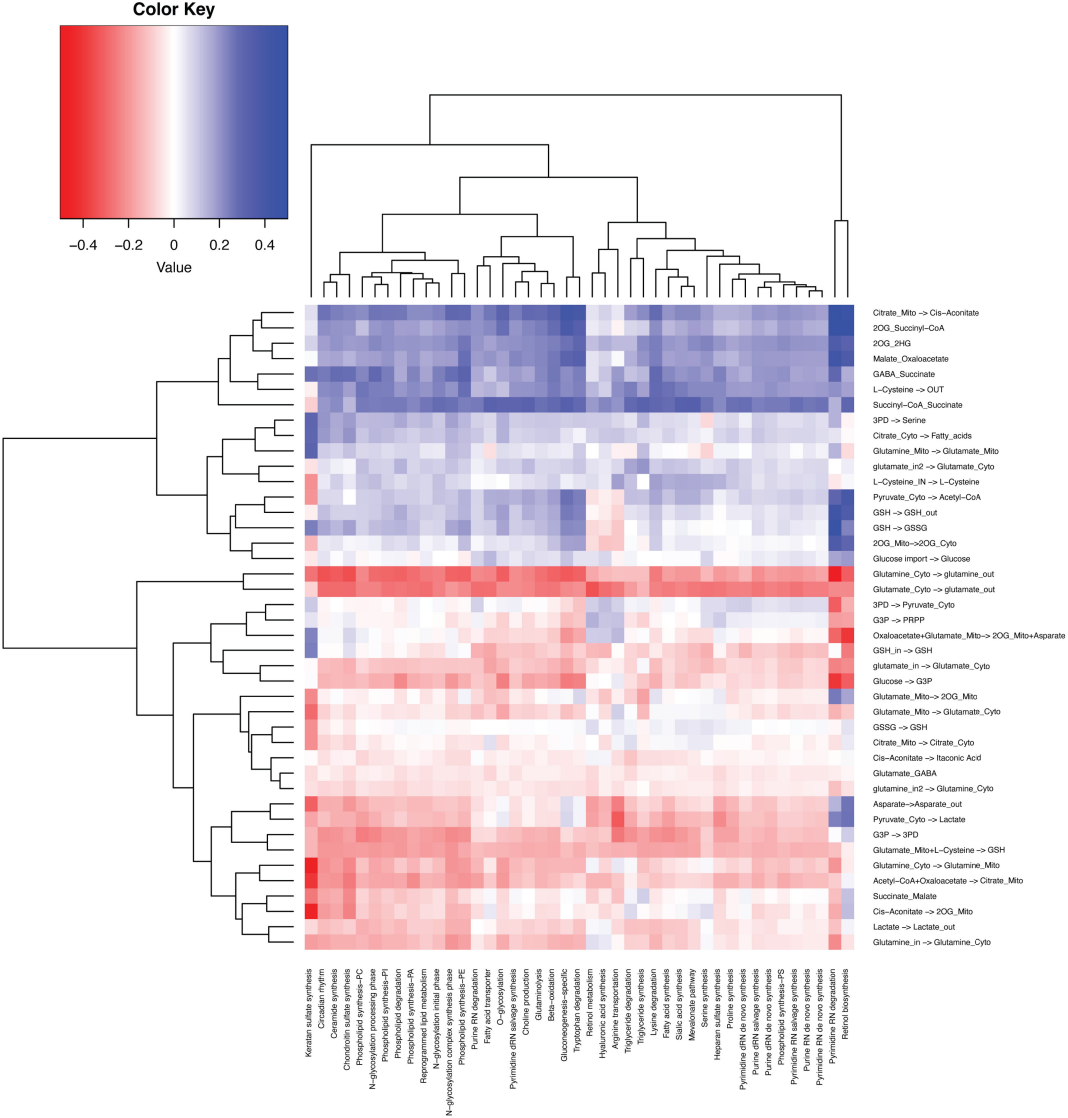
Correlation between the fluxome of the central metabolic network and the expression level of 42 metabolic stress-related modules.

Sun et al. summarized 42 metabolic stress marker gene sets (23). We also examined the correlation between sample-wise gene expression level of the metabolic stress marker gene sets computed by single-sample Gene Set Enrichment Analysis (ssGSEA) *vs*. the scFEA-predicted sample-wise fluxome (**Figure 6**). As the majority of the stress marker sets are biosynthesis-related, the decreasing of which suggests elevated metabolic stress or unmet demand. We observed that the TCA cycle, biosynthesis of amino acids and fatty acids, and the enzyme-catalyzed reaction of GSH are positively correlated with the stress marker sets, while the glycolysis, lactate production, glutaminolysis, and antioxidation role of GSH have negative correlations. Our analysis identified biosynthesis-favored (positive correlation) and unfavored (negative correlation) metabolic modules in the central metabolism network. Notably, glutaminolysis-related modules, especially the import of glutamine and glutamate are unfavored in cancer tissues with high biosynthesis, suggesting a limited role of glutaminolysis in fueling the biosynthesis of large molecules. An alternative explanation is that when redox reactions involved biosynthesis activity are suppressed, more glutamine and glutamate and their cytosolic reactions are needed by cancer cells to sustain sufficient amino acids and macromolecule biosynthesis.

## Discussion

In this study, we reported the convergence of metabolic phenotypes in the disease progression of multiple cancer types derived from a pan-cancer-level analysis of 5,253 TCGA cancer and normal samples. This result is consistent with our previous observations on single-cell RNA-seq data of the TME of melanoma and head and neck cancer, i.e., immune and stromal cells tend to have higher metabolic heterogeneity compared to cancer cells (14). While the Warburg effect has been identified as a common hallmark of almost all cancer types, heterogeneity in the means of energy production has rarely been studied, i.e., the choice between the anaerobic glycolytic pathway and aerobic TCA cycle pathway. Our observations provide new theories of the trajectories of metabolic shift that occurred in the oncogenesis process; that is, for a large set of solid cancer types, their metabolic phenotypes, determined by the flux distribution of the central metabolic pathways, tend to converge through the oncogenesis process. This indicates that it is the converged phenotype, rather than the path to convergence, that embodies the hallmark property of cancer progression. This “convergence” theory is rational, as for cancer cells, the optimal flux distribution should allow cells to sustain a fast cell proliferation rate and high fitness level under hostile and dynamic biochemical conditions in TME, which is independent of cancer types and tissue origins. This theory could also explain why metabolic inhibitors have not been very successful in cancer treatment. While different tumor tissues may converge to the same metabolic state, they may undergo different paths to such convergence. Hence, simply blocking a particular path may not stop the cancer progression to the desirable phenotype. A future direction is to comprehensively characterize the converged metabolic state that is most desirable for cancer progression, including the level of reaction rate and the ratio of energy produced using different branches. Also, new computational measures are needed to identify the trajectory of metabolic shifts for each cancer and the distance of its current metabolic phenotype to the optimal metabolic state. Our current analysis suggests that genetic mutation is not enough to explain how heterogeneous paths are formed toward an optimal metabolic phenotype. We speculate that the shifts and evolution of metabolism are triggered by the coevolution between cancer and the intra- and intercellular biochemical condition within its TME and facilitated by epigenetic regulations.

Numerous computational analyses have been proposed to study metabolic variations in cancer and other systems (24–29). However, while substantial efforts have been paid on reconstructing metabolic pathways, a fundamental question that remains unaddressed is how metabolic activities differ among cells of different morphological types, physiological states, tissues, or disease backgrounds that have the same genetic constitutions. Compared to other omics data such as metabolomics, fluxomics, and proteomics that can be applied to study metabolic reprogramming in cancer, transcriptomics data are of the highest availability. Although transcriptomics experiments have been utilized to characterize metabolic alterations in diseases (30, 31), existing analyses tend to portray the average change of intermixed and heterogeneous cell subpopulations within a given tissue (32–34). This makes it impossible to further study the metabolic heterogeneity and cell-wise flux changes in complex tissue, in which cells are well understood to rewire their metabolism and energy production in response to varied biochemical conditions (8, 35–37). Compared to other well-studied biological mechanisms, such as immuno-response or transcriptional regulatory activity, there are substantial gaps in characterizing metabolic changes using omics data with tailored systems biology models and statistical metrics.

To the best of our knowledge, our recently developed scFEA is the first and only method to estimate cell-wise metabolic flux and metabolomic changes by approximating the underlying dynamical systems models (38). Our analysis demonstrated the feasibility of deciphering the cause and impact of metabolic variations by using multi-omics data in an explicit way, namely data-driven and AI-empowered systems biology. On the other hand, in our preliminary study, we identified three major remaining challenges in leveraging the high-resolution multi-omics data and the stochiometric relations of the metabolic network that need to be solved to best characterize the dynamics and context specificity of metabolic activities: (1) *How to reconstruct disease, tissue, and cell group specific metabolic network?* A complex tissue microenvironment may be constituted by cells with different metabolic abnormalities, heterogeneous metabolic networks, varied preferences, and dependencies (39–43). Mapping multi-omics data to a common and static metabolic network precludes the discovery of hidden and dynamic relationships among the metabolic units, making it impossible to identify the key players in disease tissue or cells and to predict the vulnerability of a particular phenotype to a certain metabolic factor. (2) *How to accurately estimate metabolic flux and identify the key causes of metabolic variations by using multi-omics data? A* big gap in metabolic modeling is how to map diverse data types onto quantitative metabolic models in order to elucidate the metabolic fluxome more thoroughly and hence achieve functional characterization and accurate quantification of all levels of metabolic activities and their interactions (44–46). Although our recent progress and other studies provide a preliminary solution, no existing method can effectively handle the heterogeneity of directions of highly reversible reactions and imbalance of intermediate metabolites among cells within a disease microenvironment. (3) *How to comprehensively define and assess the mechanisms and representation forms of metabolic variations on multi-omics data?* Metabolic variations happen on different levels, such as genes, enzymes, metabolites, network structure, or flux (kinetic models). How to design valid metrics and statistical models to quantify the true impact of such variations on context-specific metabolic activity remains unsolved.

As noted, our study and method demonstrated a prototype of a new research direction, namely “data-driven and AI-empowered systems biology.” For given omics data and a to-be-studied biological process, the underlying goal is to identify a mathematical model that could not only quantify the biological process but also approximate its dynamic property over the data. The established model should leverage the coherency of the physical or chemical laws of the system and the goodness of fitting the data. Compared to the conventional differential equation-based systems biology model, our approach does not rely on the preassessed kinetic parameter and is therefore not limited by the reductionist paradigm that can be applied to characterize a relatively large system, such as the central metabolism network, in complex disease.

## Methods

### Data used in this study

#### TCGA transcriptomic data

TCGA RNA-seq v2 FPKM data of the nine cancer types (11 subtypes) were retrieved from the Genomic Data Commons (GDC) data portal using TCGAbiolinks (47). Log(FPKM+1)-normalized gene values were utilized for flux estimation. Clinical data were obtained in XML format from GDC and parsed with an in-house script. GENCODE gene annotations used by the GDC data processing pipeline were downloaded directly from the GDC reference file webpage.

### Validation datasets

#### GTEx data

GTEx RNA-seq data and sample information (48) were downloaded *via the* Xena browser. Log(FPKM+1)-normalized gene values were utilized for flux estimation. We only included the tissue types that had at least 20 samples.

#### CCLE data

CCLE RNA-seq data and sample information (49) were downloaded from DepMap. Log(FPKM+1)-normalized gene values were utilized for flux estimation. We only included the tissue origins that have at least 20 cell line types.

#### GSE72056 scRNA-seq data

GSE72056 dataset (50) is collected from human melanoma tissues. The original paper provided cell classification and annotations, including B cells, cancer-associated fibroblast (CAF) cells, endothelial cells, macrophage cells, malignant cells, NK cells, T cells, and unknown cells.

#### GSE103322 scRNA-seq data

GSE103322 dataset (51) is collected from head and neck cancer tissues. The original paper provided cell classification and annotations, including B cells, dendritic cells, endothelial cells, fibroblast cells, macrophage cells, malignant cells, mast cells, myocyte cells, and T cells. Notably, as indicated by the original work, malignant cells have high intertumoral heterogeneity.

#### Pa03c scRNA-seq data (GSE173433)

We have previously collected Pa03c scRNA-seq data (19) of 40 APEX1 knockdown (KD) and 48 scrambled control cells of Pa03c patient-derived pancreatic cancer cell line under hypoxia condition. We also collected metabolomic profiling of selected metabolites of APEX1 KD and scrambled control Pa03c cells under hypoxia by using S-1 Mitoplates (Biolog, Hayward, CA, USA).

### Main methods

#### scFEA

We have directly applied our scFEA method to the TCGA and the two scRNA-seq data against the iron metabolic map. While the details of the method are given in Alghamdi et al. (14), we outline the key ideas of the algorithm. The inputs to scFEA are gene expression data and a factor graph-based representation of the metabolic map. Let $\text{FG}\left(C^{1\times K},RM^{1\times M},\;E=\;\left\{E_{C\to R},E_{R\to C}\right\}\right)$ be a given factor graph, where $C^{1\times K}=\left\{C_{k},k=1,\ldots ,K\right\}$ is the set of K metabolites, $RM^{1\times M}=\left\{R_{m},m=1,\ldots ,M\right\}$ the set of M metabolic reactions (represented as a rectangle in **Figure 1A**), $E_{C\to R}\text{ and }E_{R\to C}$ represent direct edges from the reaction $R_{m}$ to metabolite $C_{k}$ and from metabolite $C_{k}\;\text{t}$ o reaction $R_{m}$ , respectively. For the k th metabolite $C_{k}$ , define the set of reactions consuming and producing $C_{k}$ as $F_{in}^{C_{k}}=\{R_{m}|(R_{m}\to C_{k})\in E_{C\to R}\}\text{ and }F_{out}^{C_{k}}=\{R_{m}|\left(C_{k}\to R_{m}\right)\in E_{R\to C}\;\}$ , which is derived from the stoichiometric matrix of the given metabolic map. For an RNA-seq dataset with N cells, denote ${\text{Flux}}_{m,j}$ as the flux of the mth reaction in the cell j, j=1…N , and $F_{j}=\left\{{\text{Flux}}_{1,j},\ldots ,{\text{Flux}}_{M,j}\right\}$ as the whole set of the reaction fluxes. Denote $G^{m}=\left\{G_{1}^{m},\ldots ,G_{i_{m}}^{m}\right\}$ as the genes associated with the reactions in $R_{m}$ , and $G_{j}^{m}=\left\{G_{i_{1},j}^{m},\ldots ,G_{i_{m},j}^{m}\right\}$ as their expressions in sample j , where $i_{m}$ is for the number of genes in $R_{m}$ .

We model ${\text{Flux}}_{m,j}=f_{nn}^{m}\left(G_{j}^{m}|\;\theta_{m}\right)$ as a multi-layer fully connected neural network with the input $G_{j}^{m}$ , where $\theta_{m}$ represents the parameters of the neural network. Then the $\theta_{m}$ **and** cell-wise flux ${\text{Flux}}_{m,j}$ can be solved by minimizing the following loss function L , where λ  serves as a hyperparameter:


$$\begin{array}{c} L=\sum_{j=1}^{N}\sum_{k=1}^{K}{\left(\sum_{m\in F_{in}^{C_{k}}}{\text{Flux}}_{m,j}-\sum_{m^{\prime }\in F_{out}^{C_{k}}}{\text{Flux}}_{m^{\prime },j}\right)}^{2}\\ +\sum_{j=1}^{N}\sum_{m=1}^{M}{\left({\text{Flux}}_{m,j}-\left|{\text{Flux}}_{m,j}\right|\right)}^{2}\\ +\lambda \sum_{j=1}^{N}{\left(\sum_{m=1}^{M}{\text{Flux}}_{m,j}-{\text{TA}}_{j}\right)}^{2} \end{array}$$

where ${\text{TA}}_{j}$ is a surrogate for the total metabolic level of cell j , which is assigned to a constant or total expression of all the metabolic genes in j . As tissue transcriptomics data are always dense, we only include three loss terms in scFEA that are sufficient to predict fluxome. The three loss terms in *L* from left to right are (1) loss of flux imbalance, (2) loss of non-negative prediction, and (3) loss to control nonzero trivial solution. As noted, the fundamental assumptions of scFEA regarding nonlinearity relations of transcriptome and metabolome and minimizing flux balance are still held in bulk RNA-seq data. In this study, we modified scFEA by only removing a loss term designed for highly sparse input and removing the data imputation step. We do not consider this modification a significant methodology-level novelty. The application of scFEA to bulk RNA-seq data has already been demonstrated in our previous work. In this study, we carefully examined the convergence of the loss function of scFEA on different inputs. scFEA achieved a good convergence in all the analyzed bulk tissue/cell and single-cell data (52). **Supplementary Figure S4** showcases the convergency of the loss function when applying scFEA to different datasets.

#### tSNE analysis

tSNE analysis was conducted using Rtsne v0.16 R package against a full fluxome profile with default parameters.

#### K-nearest-neighbor clustering

K-nearest-neighbor clustering was conducted using the Seurat v3 R package against the top 12 principal components. The number of clusters is determined by default settings in the Seurat package (53).

#### ssGSEA

We applied the ssGSEA2.0 R package to estimate the levels of the selected RMs on individual samples (54, 55). The ES score computed by ssGSEA was utilized to represent the level of each RM. Gene sets of the RMs were collected and annotated in our previous work (14).

#### Statistical test of differential analysis

We have utilized the Mann–Whitney *U* test for all differential analyses, including differential gene expression analysis and the difference in predicted flux. We utilized *p*-value of< 0.001 as a significant cutoff for the multiple differential tests of metabolic flux and abundance.

## Data availability statement

The datasets presented in this study can be found in online repositories. The names of the repository/repositories and accession number(s) can be found in the article/**Supplementary Material**.

## Author contributions

HZ, SC, and CZ proposed and supervised the project. GY, SH, KH, AL, JY, NM, PD, and YW conducted data collection, processing, and analysis. GY, SH, KH, and AL conducted result summaries, visualizations, and validations. All authors contributed to the manuscript’s development. The major analysis by GY, SH, KH, AL, JY, and NM was conducted within the 2021 SEED and STEM summer research program and the 2022 IUSM CCBB summer research internship. Parental consent was obtained to participate as a researcher in the study. All the authors were above 16 when the manuscript was submitted.
