# Supplementary material for: Flux estimation analysis systematically characterizes the metabolic shifts of the central metabolism pathway in human cancer
Source: Front Oncol. 2023 Jun 12;13:1117810. doi: 10.3389/fonc.2023.1117810 (PMC10291142; doi:10.3389/fonc.2023.1117810)
Supplement: Supplementary file 1 [file DataSheet_1.pdf]

SUPPLEMENTARY FIGURES

Supplementary Figure S1. (A) Metabolomics profile of selected metabolites measured by mitochondria assay. (B) Metabolomics changes of selected metabolites predicted by scFEA in the Pa03c data. The y-axis represents the predicted metabolomic change, with positive and negative values suggesting that the metabolites are more likely to be accumulated or depleted in the corresponding condition. The x-axis represents the cells of scrambled control (sc_h) and APEX1-knockdown (si_h) under hypoxia condition.

Supplementary Figure S2. Predicted flux for all the 42 reaction modules in cancer (red) vs normal (blue).

Supplementary Figure S3. Distribution of samples of each cancer and normal tissue type in the tSNE plot (associated with Figure 4A).

Supplementary Figure S4. The convergence of loss terms when applying scFEA to different datasets.

SUPPLEMENTARY TABLES

Supplementary Table S1. P values and mean difference of the predicted metabolic flux and metabolic abundance in cancer vs normal in the 11 analyzed TCGA cancer types.

Supplementary Table S2. Information of the reaction modules, reactions, and compounds; Predicted flux of each module in each sample; Predicted metabolomic variations (tend of accumulation or depletion) of each intermediate compound in each sample.

Supplementary Table S3. Distribution of cancer and normal tissue types in each identified cluster (associated with Figure 4B).
