# Supplementary figures and images for: Flux estimation analysis systematically characterizes the metabolic shifts of the central metabolism pathway in human cancer
Source: Front Oncol. 2023 Jun 12;13:1117810. doi: 10.3389/fonc.2023.1117810 (PMC10291142; doi:10.3389/fonc.2023.1117810)

**A**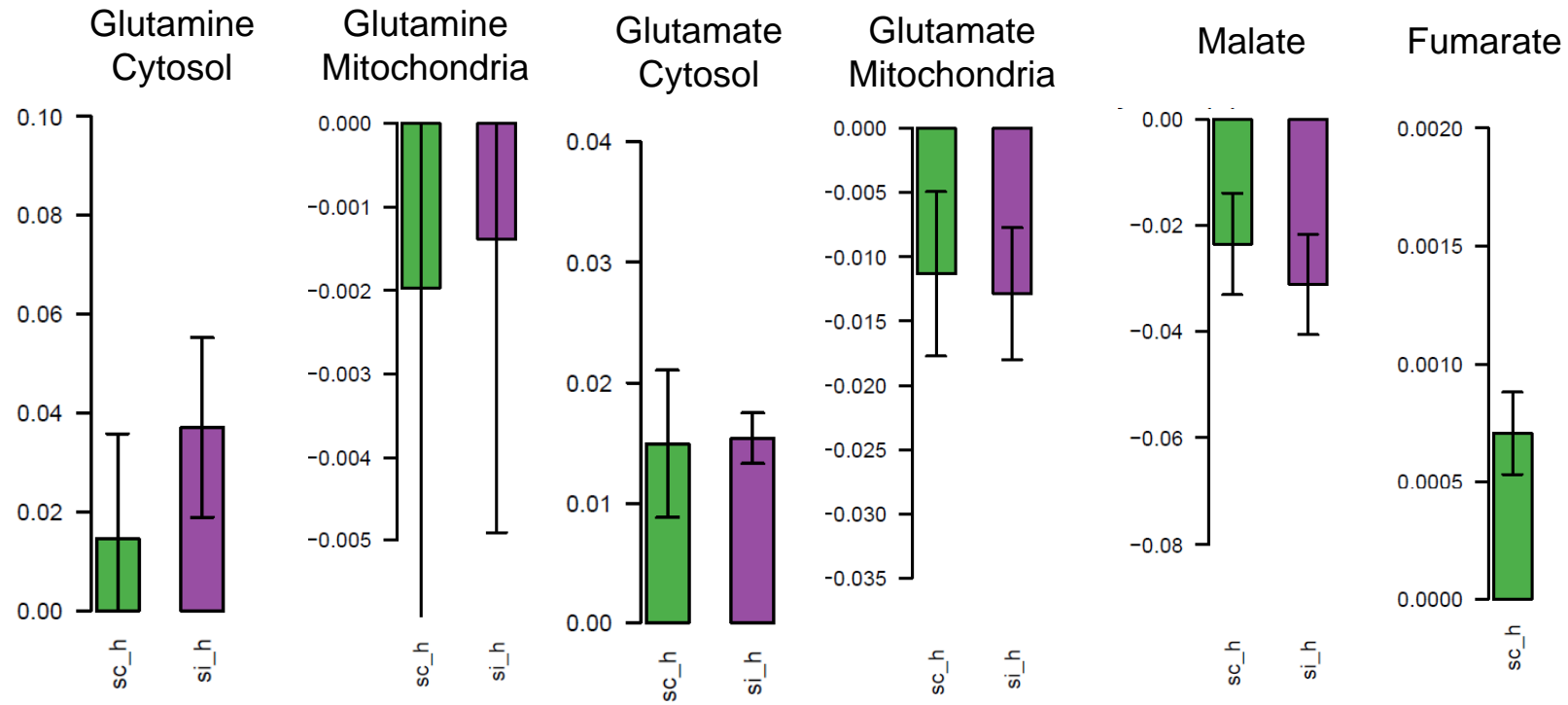**B**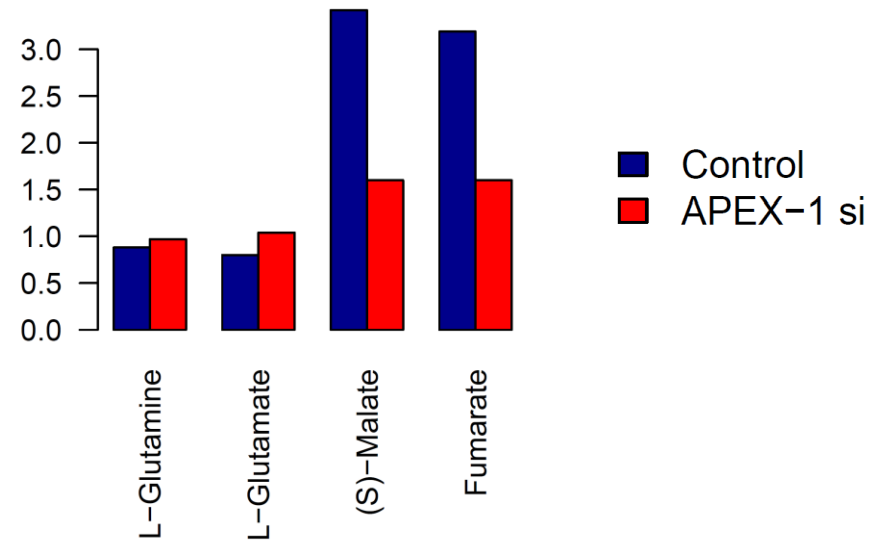

Supplement: Supplementary file 2 [file DataSheet_2.pdf]
