# Supplementary material for: Flux estimation analysis systematically characterizes the metabolic shifts of the central metabolism pathway in human cancer
Source: Front Oncol. 2023 Jun 12;13:1117810. doi: 10.3389/fonc.2023.1117810 (PMC10291142; doi:10.3389/fonc.2023.1117810)
Supplement: Supplementary file 3 [file DataSheet_3.pdf]

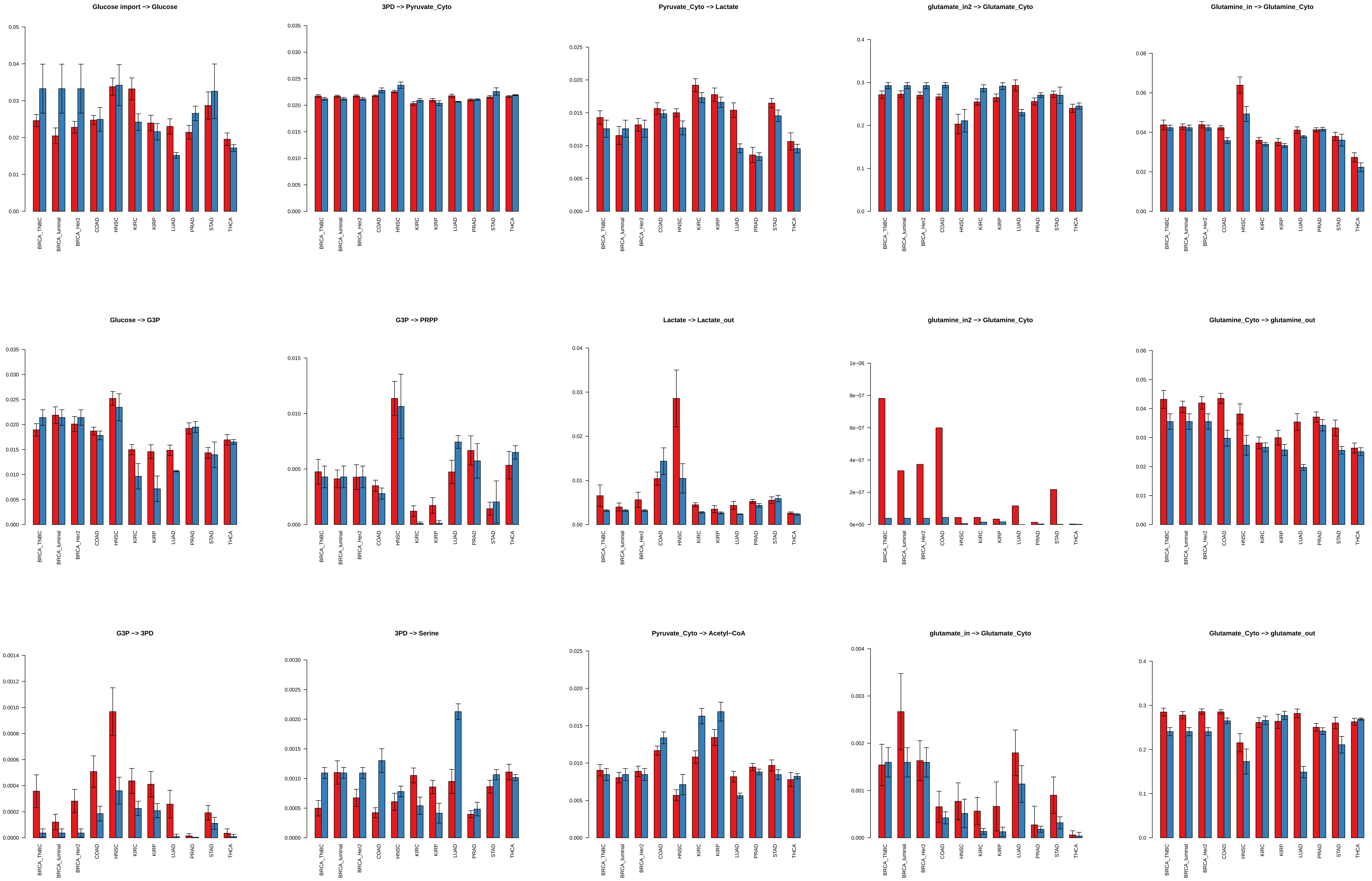
Glucose import -> Glucose
0.00 0.01 0.02 0.03 0.04 0.05
3PD -> Pyruvate_Cyto
0.000 0.005 0.010 0.015 0.020 0.025 0.030 0.035
Pyruvate_Cyto -> Lactate
0.000 0.005 0.010 0.015 0.020 0.025
glutamate_in2 -> Glutamate_Cyto
0.0 0.1 0.2 0.3 0.4
Glutamine_in -> Glutamine_Cyto
0.00 0.02 0.04 0.06 0.08
Glucose -> G3P
0.000 0.005 0.010 0.015 0.020 0.025 0.030 0.035
G3P -> PRPP
0.000 0.005 0.010 0.015
Lactate -> Lactate_out
0.00 0.01 0.02 0.03 0.04
glutamine_in2 -> Glutamine_Cyto
0e+00 2e-07 4e-07 6e-07 8e-07 1e-06
Glutamine_Cyto -> glutamine_out
0.00 0.01 0.02 0.03 0.04 0.05 0.06
G3P -> 3PD
0.0000 0.0002 0.0004 0.0006 0.0008 0.0010 0.0012 0.0014
3PD -> Serine
0.0000 0.0005 0.0010 0.0015 0.0020 0.0025 0.0030
Pyruvate_Cyto -> Acetyl-CoA
0.000 0.005 0.010 0.015 0.020 0.025
glutamate_in -> Glutamate_Cyto
0.000 0.001 0.002 0.003 0.004
Glutamate_Cyto -> glutamate_out
0.0 0.1 0.2 0.3 0.4
BRCA_TNBC BRCA_luminal BRCA_Her2 COAD HNSC KIRC KIRP LUAD PRAD STAD THCA

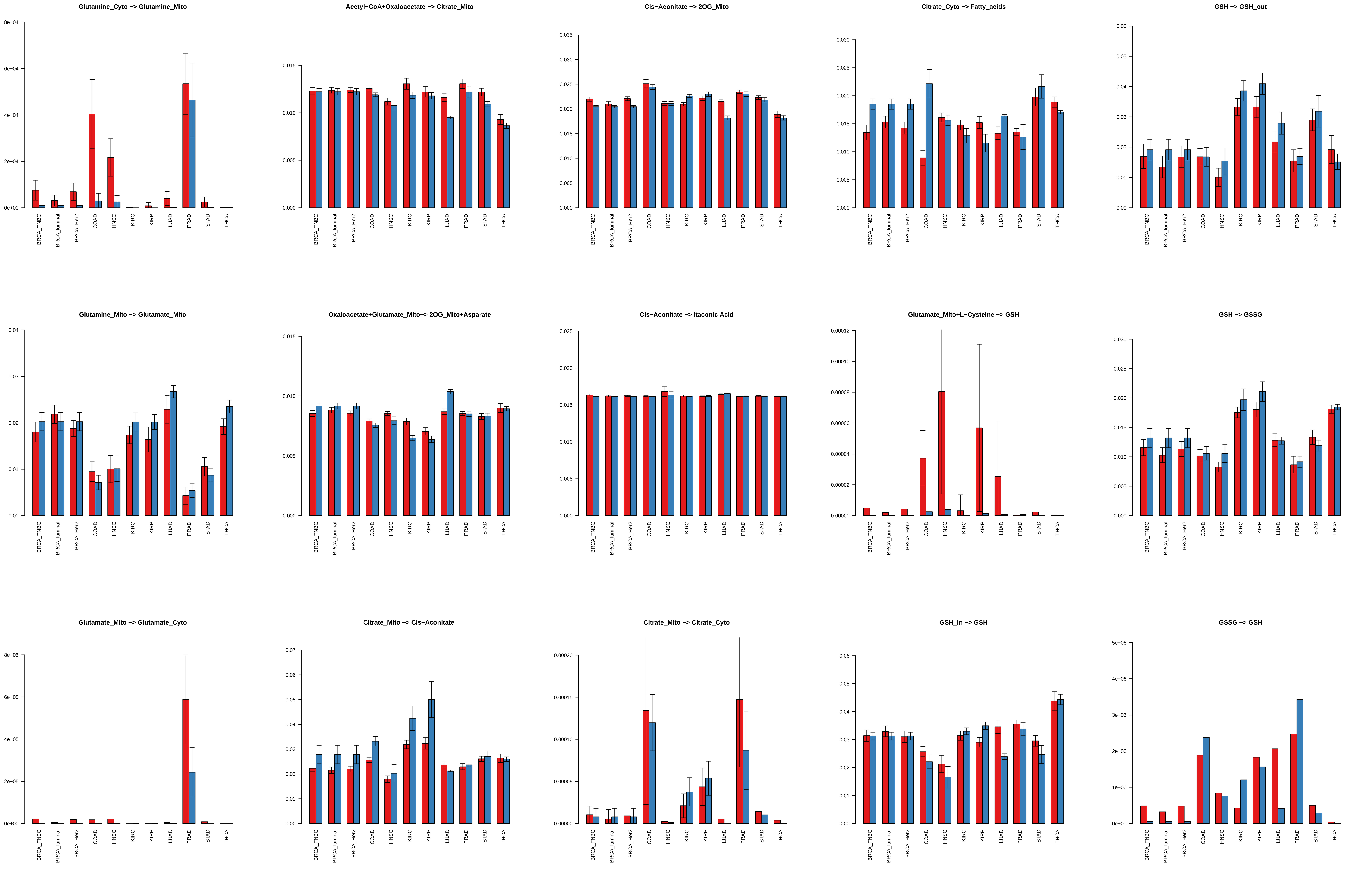

Glutamine_Cyto −> Glutamine_Mito
0e+00 2e−04 4e−04 6e−04 8e−04
Acetyl−CoA+Oxaloacetate −> Citrate_Mito
0.000 0.005 0.010 0.015
Cis−Aconitate −> 2OG_Mito
0.000 0.005 0.010 0.015 0.020 0.025 0.030 0.035
Citrate_Cyto −> Fatty_acids
0.000 0.005 0.010 0.015 0.020 0.025 0.030
GSH −> GSH_out
0.00 0.01 0.02 0.03 0.04 0.05 0.06
Glutamine_Mito −> Glutamate_Mito
0.00 0.01 0.02 0.03 0.04
Oxaloacetate+Glutamate_Mito−> 2OG_Mito+Asparate
0.000 0.005 0.010 0.015
Cis−Aconitate −> Itaconic Acid
0.000 0.005 0.010 0.015 0.020 0.025
Glutamate_Mito+L−Cysteine −> GSH
0.00000 0.00002 0.00004 0.00006 0.00008 0.00010 0.00012
GSH −> GSSG
0.000 0.005 0.010 0.015 0.020 0.025 0.030
Glutamate_Mito −> Glutamate_Cyto
0e+00 2e−05 4e−05 6e−05 8e−05
Citrate_Mito −> Cis−Aconitate
0.00 0.01 0.02 0.03 0.04 0.05 0.06 0.07
Citrate_Mito −> Citrate_Cyto
0.00000 0.00005 0.00010 0.00015 0.00020
GSH_in −> GSH
0.00 0.01 0.02 0.03 0.04 0.05 0.06
GSSG −> GSH
0e+00 1e−06 2e−06 3e−06 4e−06 5e−06
BRCA_TNBC BRCA_luminal BRCA_Her2 COAD HNSC KIRC KIRP LUAD PRAD STAD THCA

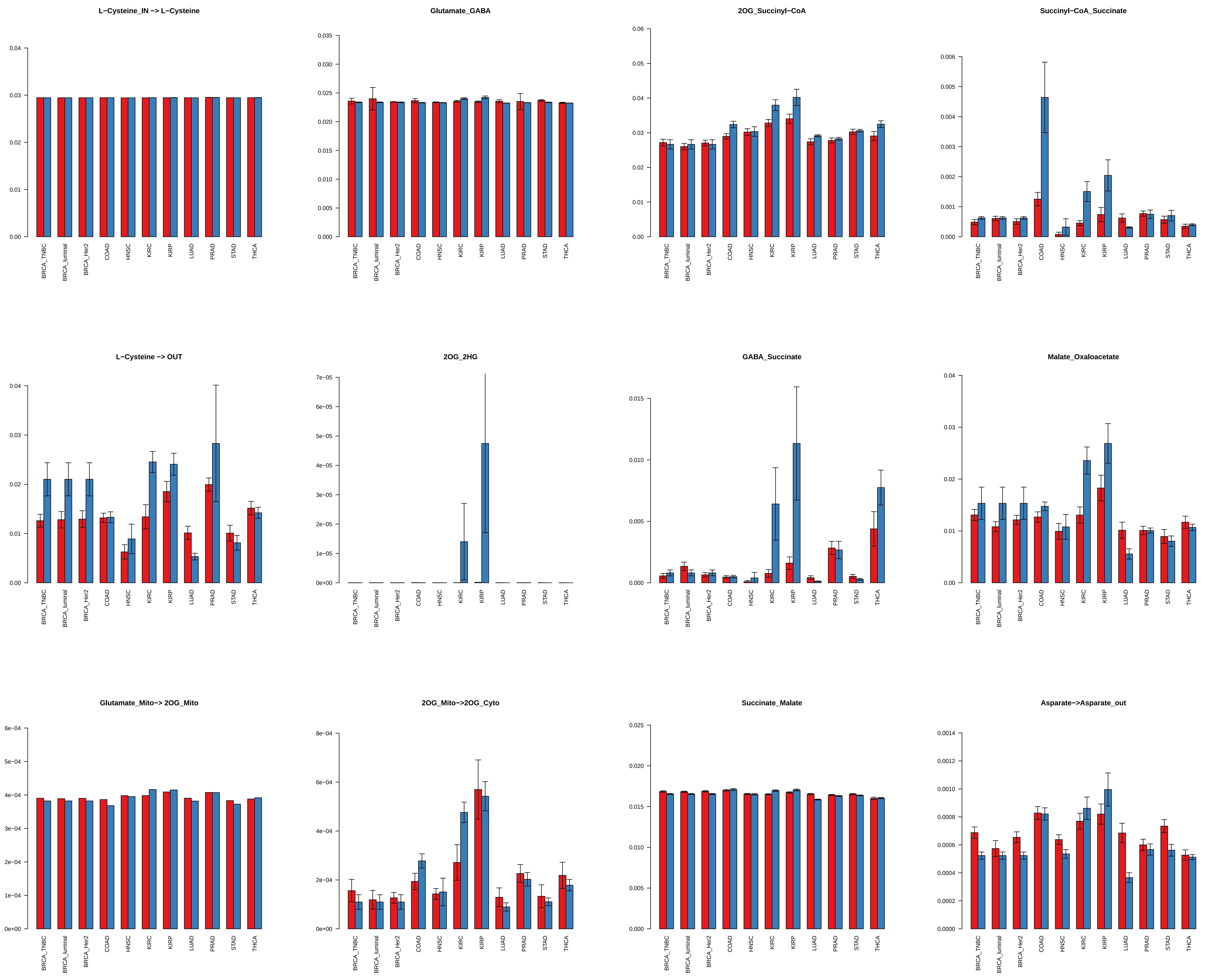

L−Cysteine_IN −> L−Cysteine
Glutamate_GABA
2OG_Succinyl−CoA
Succinyl−CoA_Succinate
L−Cysteine −> OUT
2OG_2HG
GABA_Succinate
Malate_Oxaloacetate
Glutamate_Mito−> 2OG_Mito
2OG_Mito−>2OG_Cyto
Succinate_Malate
Asparate−>Asparate_out
BRCA_TNBC
BRCA_luminal
BRCA_Her2
COAD
HNSC
KIRC
KIRP
LUAD
PRAD
STAD
THCA
