# Supplementary material for: Flux estimation analysis systematically characterizes the metabolic shifts of the central metabolism pathway in human cancer
Source: Front Oncol. 2023 Jun 12;13:1117810. doi: 10.3389/fonc.2023.1117810 (PMC10291142; doi:10.3389/fonc.2023.1117810)
Supplement: Supplementary file 4 [file DataSheet_4.pdf]

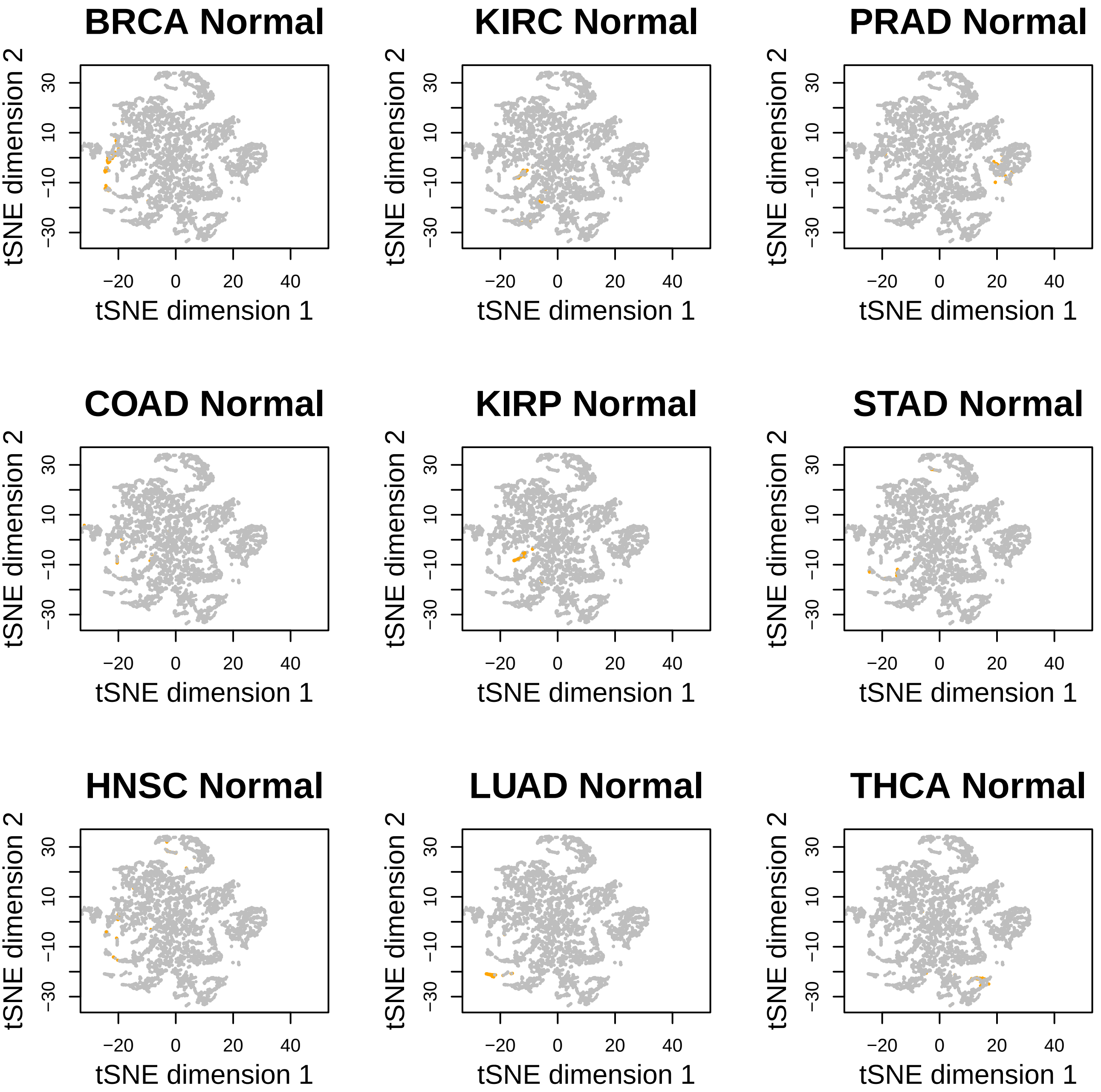

BRCA Normal
KIRC Normal
PRAD Normal
COAD Normal
KIRP Normal
STAD Normal
HNSC Normal
LUAD Normal
THCA Normal
tSNE dimension 1
tSNE dimension 2
-20
0
20
40
-30
-10
10
30

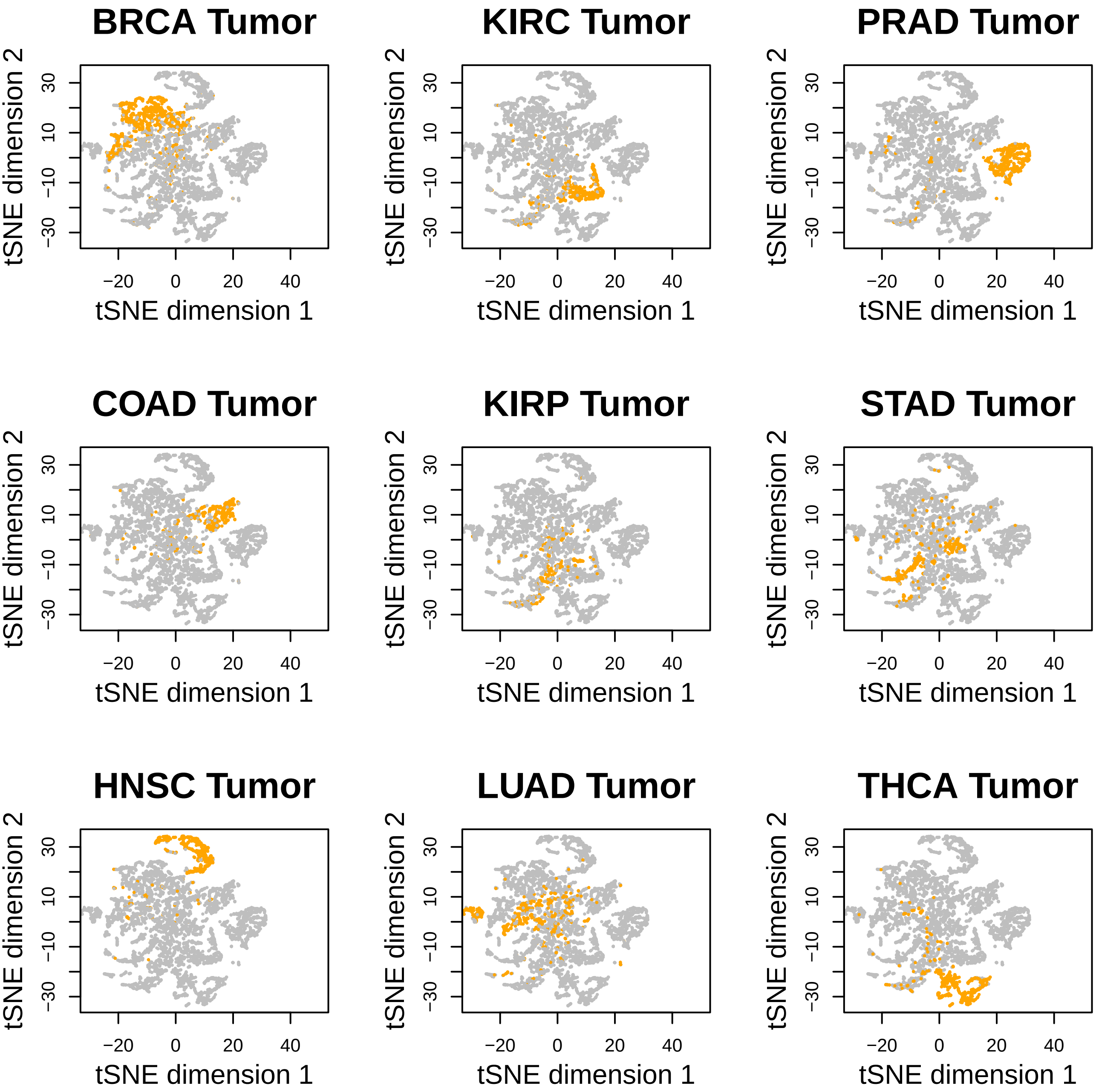
BRCA Tumor
KIRC Tumor
PRAD Tumor
COAD Tumor
KIRP Tumor
STAD Tumor
HNSC Tumor
LUAD Tumor
THCA Tumor
tSNE dimension 1
tSNE dimension 2
−20
0
20
40
30
10
−10
−30
