# Supplementary material for: Flux estimation analysis systematically characterizes the metabolic shifts of the central metabolism pathway in human cancer
Source: Front Oncol. 2023 Jun 12;13:1117810. doi: 10.3389/fonc.2023.1117810 (PMC10291142; doi:10.3389/fonc.2023.1117810)
Supplement: Supplementary file 5 [file DataSheet_5.pdf]

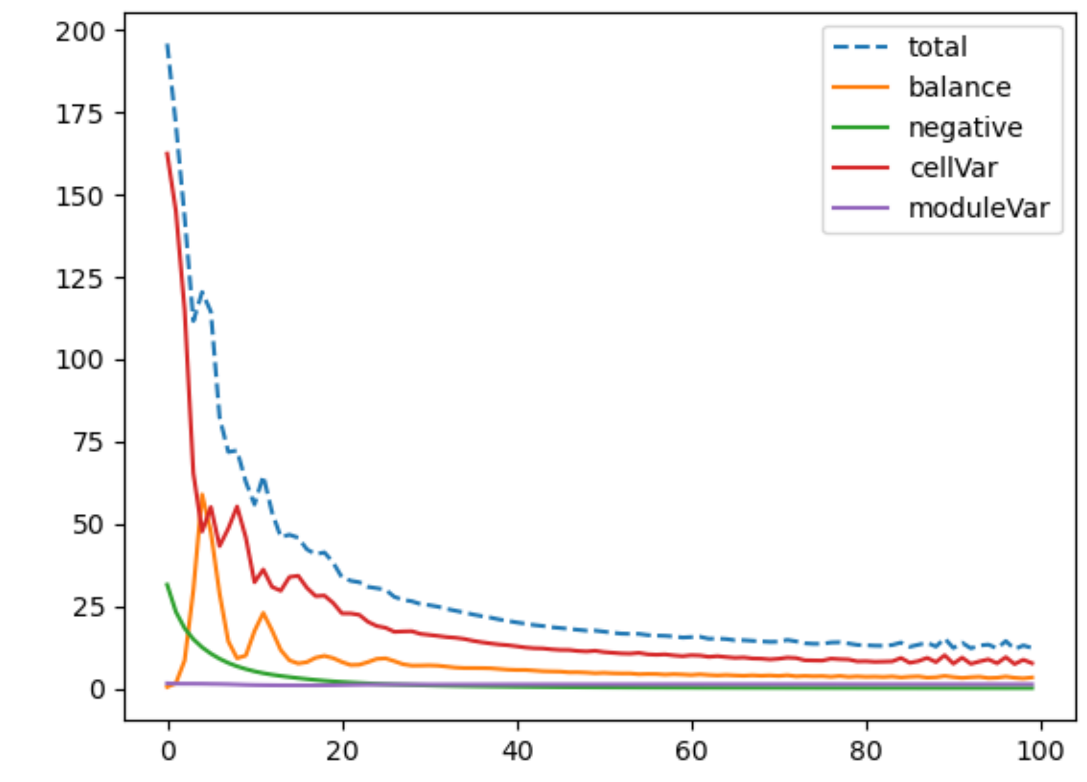

Pa03c

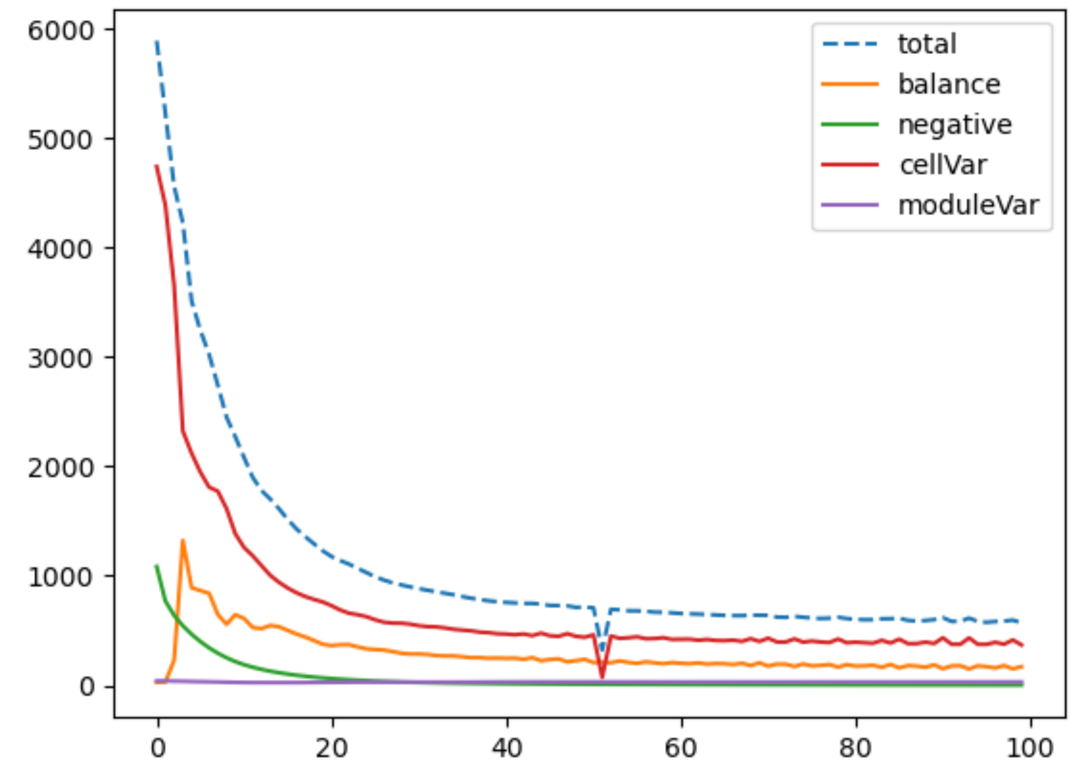

GSE72056

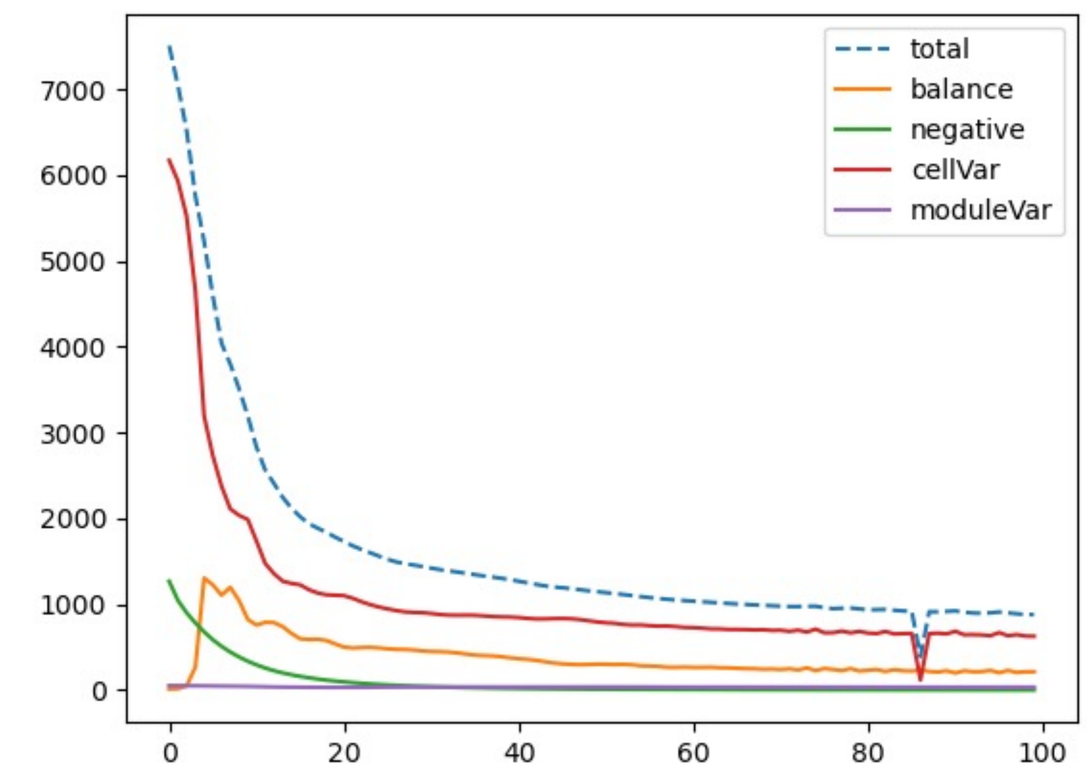

GSE103322

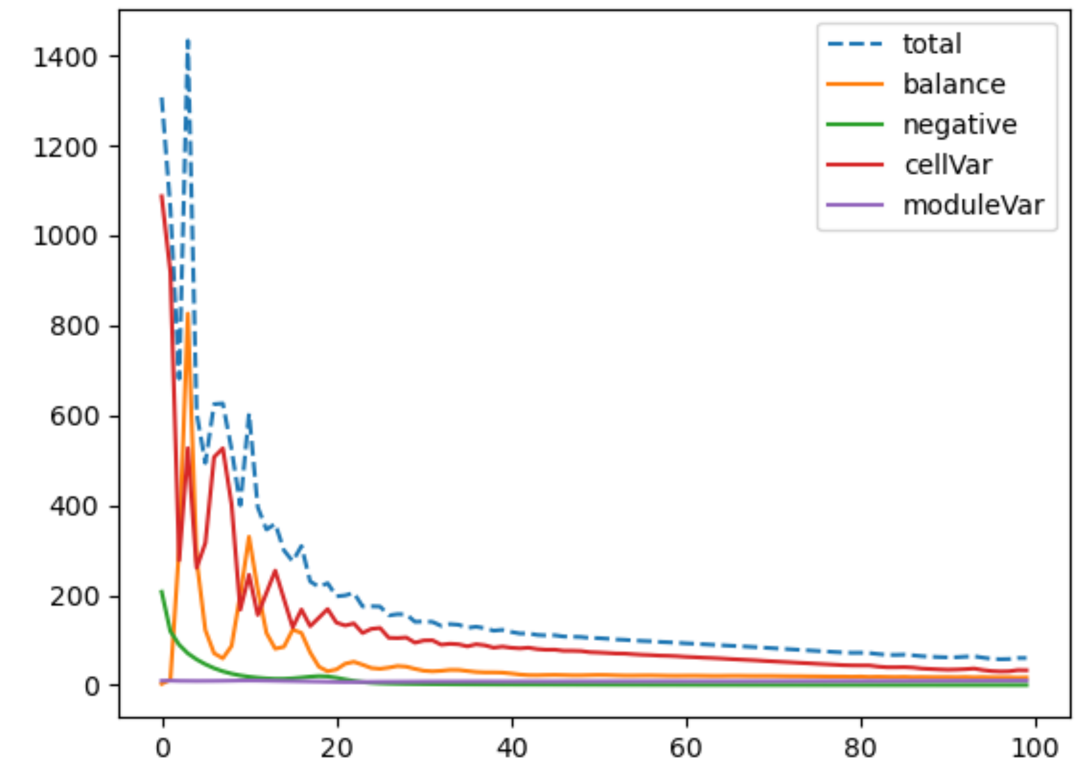

CCLE

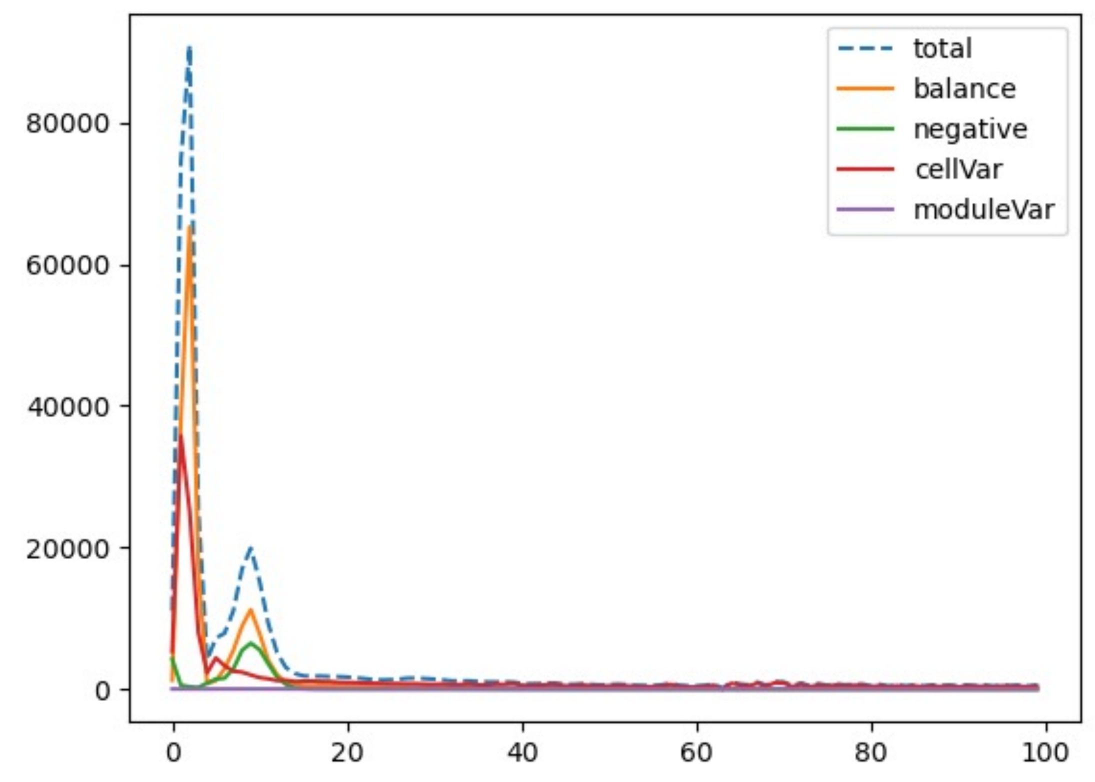

GTEX

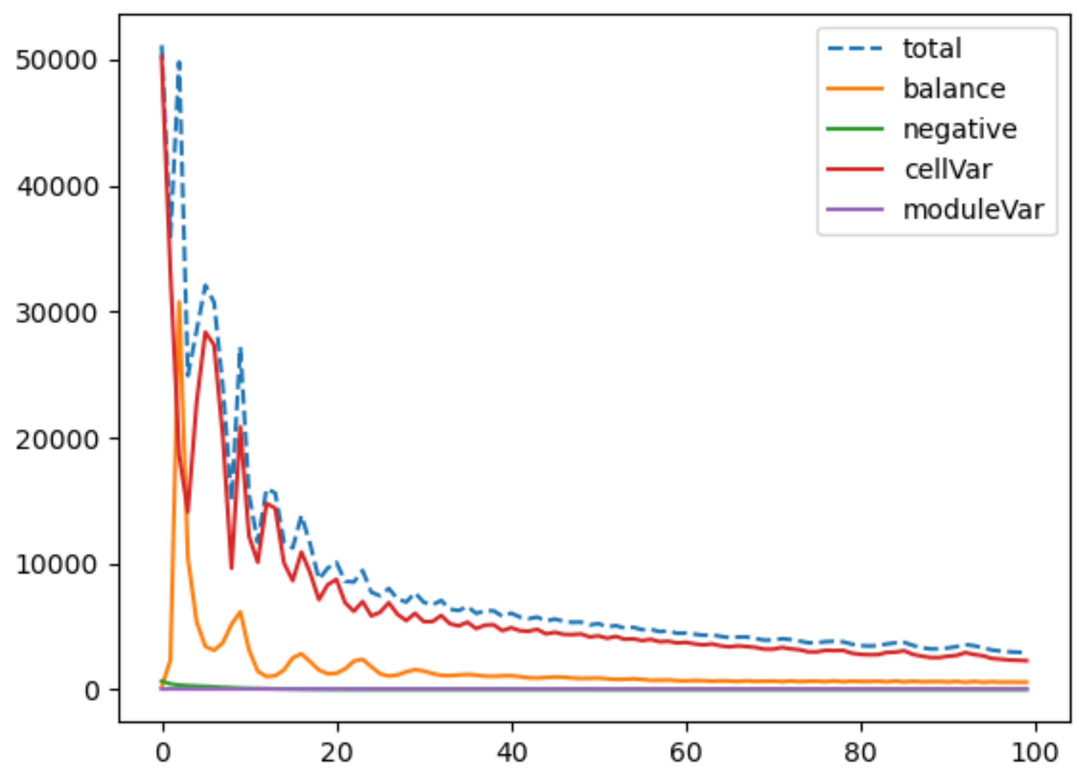

TCGA

Supplementary Figure S4: The convergence of loss terms when applying scFEA to different datasets
